# 新型BTK抑制剂治疗慢性淋巴细胞白血病临床研究进展

**DOI:** 10.3760/cma.j.issn.0253-2727.2023.11.015

**Published:** 2023-11

**Authors:** 彤璐 邱, 祎 缪, 建勇 李

**Affiliations:** 南京医科大学第一附属医院，江苏省人民医院血液科，南京医科大学血液研究重点实验室，江苏省肿瘤个体化医学协同创新中心，南京 210029 Department of Hematology, Key Laboratory of Nanjing Medical University, the First Affiliated Hospital of Nanjing Medical University, Jiangsu Province Hospital, Collaborative Innovation Center for Cancer Personalized Medicine, Nanjing 210029, China

慢性淋巴细胞白血病（CLL）是老龄化社会常见的血液肿瘤，在西方国家为成人最常见的白血病，而随着我国人口老龄化速度加快，我国的CLL患者人数出现上升趋势[1]。B细胞表面受体（BCR）介导的信号通路在CLL的发病中起重要作用，调控细胞生长、增殖、分化和黏附等关键细胞功能。布鲁顿酪氨酸激酶（BTK）是BCR信号通路关键组成部分，在CLL中呈高表达状态，BTK抑制剂（BTKi）的成功研发，为治疗CLL带来了突破性进展。伊布替尼（Ibrutinib）为首个获批用于治疗CLL的BTKi，通过与BTK活性部位481位置的半胱氨酸残基形成共价键抑制BTK活性，阻断BCR多个下游信号通路达到治疗效果[2]。多个临床试验的长期随访结果表明伊布替尼可以显著延长复发/难治CLL（R/R CLL）患者和初治CLL（TN CLL）患者的无进展生存（PFS）期与总生存（OS）期，且安全性显著优于化学免疫治疗，但同时也暴露了伊布替尼的一些局限性。首先伊布替尼对BTK的选择性欠佳，对与BTK结构相似的EGFR、ITK和TEC同样有抑制作用，从而导致“脱靶效应”。多个临床试验和真实世界研究结果表明伊布替尼治疗中后期增加房颤和高血压等心血管不良事件风险，并且发生率持续增长，是临床应用中造成患者停药的重要原因[3]–[4]。第二代BTKi如阿可替尼（Acalabrutinib）、泽布替尼（Zanubrutinib）和奥布替尼（Orelabrutinib），通过对药物的分子结构进行优化后在临床前研究以及部分Ⅰ期临床试验中展现出了较伊布替尼更高的选择性以及更强的药效（表1）。第二代BTKi对与BTK结构相似的激酶抑制程度明显减少，对BTK占有率更高，增加单药治疗缓解深度以及联合治疗成功概率[5]。已有的系列临床试验表明第二代BTKi在药效与安全性上均有一定优势。但部分CLL患者长期临床引用共价结合BTKi后会出现耐药问题，其中最可能的耐药机制是其结合位点突变。最常见的是半胱氨酸突变为丝氨酸（BTK C481S）或突变为精氨酸（BTK C481R），导致无法形成共价键并产生优势克隆，最终出现耐药。同时受限于药物半衰期，用药间隔期间BTK不能完全受到抑制也可能促进耐药问题产生[6]。可逆非共价结合BTKi和BTK靶向蛋白水解嵌合体（BTK PROTAC）有望解决共价结合型BTKi的耐药问题，为共价结合型BTKi治疗失败的CLL带来希望。本文综述第二代BTKi治疗CLL临床试验研究结果，以及可逆非共价结合BTKi和BTK PROTAC临床进展，为临床治疗CLL提供参考。

**表1:** 不同布鲁顿酪氨酸激酶（BTK）抑制剂药效学和靶点选择性比较[5]

药名	药效			选择性		
	K_inact_/K_I_（M^−1^s^−1^）	BTK靶点抑制率（％）	体内半衰期（h）	整体选择性（％）	结构相似[IC50/EC50（nmol/L）]	
					TEC	ITK
伊布替尼	（4.77±1.67）×10^5^	96％～99％	4~13	9.4	10±12	4.9±1.2
阿可替尼	（3.11±1.48）×10^4^	99％	1	1.5	126±11	>1 000
泽布替尼	（2.79±5.89）×10^5^	淋巴结中100％	4	4.3	44±19	50±5
奥布替尼	150 mg/次，1次/d给药剂量，对患者外周血单核细胞（PBMC）BTK靶点24 h内接近100％持续抑制			456种激酶的选择性检测中，1 µmol/L奥布替尼仅对BTK的抑制作用超过90%，对其他激酶没有明显抑制作用		

注 K_incat_：激酶最大抑制率；K_I_：达激酶最大抑制率50％时抑制剂浓度；BTK靶点抑制率：服药后4 h评估患者外周血单核细胞（PBMC）BTK靶点结合水平；整体选择性：KINOMEscan 平台测定1 µmol药物对除BTK外有抑制作用的激酶占全体比值；IC50：半抑制浓度即抑制50%生物学效应所需要的药物浓度；EC50：半最大效应浓度即能引起50％最大效应的药物浓度；TEC：肝细胞癌表达的酪氨酸激酶，*x±s*；ITK：IL-2诱导T细胞激酶，*x±s*

一、第二代共价结合型高选择性BTKi单药治疗临床试验

1. 阿可替尼（ACP-196）：阿可替尼早期临床试验包括Ⅰ/Ⅱ期的ACE-CL-001试验和Ⅱ期的NCT02337829。ACE-CL-001受试者包括R/R CLL、TN CLL以及伊布替尼不耐受患者。中位随访4年后R/R CLL和TN CLL患者的总缓解率（ORR）分别为94％和97％，各亚组患者之间差异无统计学意义，伴del（17p）患者ORR可达100％。安全性方面，不良事件（AE）种类和伊布替尼基本类似，但心血管事件和高血压发生率明显降低[7]–[8]。对于伊布替尼不耐受患者，中位随访19个月ORR为71％。与伊布替尼不耐受相关的AE中72％没有再次出现，13％ AE再发但程度减轻[9]。Ⅱ期的NCT02337829[10]研究重点关注了阿可替尼的药代动力学以及BTK占有率。研究中对20例患者治疗前、治疗第1周期后和第6周期后对外周血纯化肿瘤细胞以及淋巴结活检样本进行了RNA测序，结果表明外周血和淋巴结中肿瘤细胞BTK通路显著下调，与肿瘤微环境相关基因的表达也同样受到抑制，稳定且持续的高BTK占有率可加强抑制作用。ACE-CL-208[11]这项Ⅱ期临床试验受试者为因严重或持续无法缓解的AE而不能耐受伊布替尼的CLL患者。中位随访35个月后48％患者仍在接受阿可替尼治疗，40％的患者出现了伊布替尼治疗期间相同的AE，其中67％的患者程度变轻。ASCEND试验[12]是一项阿可替尼、艾代拉里塞联合利妥昔单抗（idelalisib with rituximab，I-R）和苯达莫司汀联合利妥昔单抗（bendamustine with rituximab，B-R）对比治疗R/R CLL的临床试验。中位随访16.1个月，阿可替尼组的中位PFS期未达到，明显好于后二者的16.5个月和15.8个月，伴del（17p）和del（11q）的患者受益更大，缓解持续时间更长。阿可替尼出现≥3级AE和因AE终止治疗的情况次数较接受I-R治疗的患者少。

2. 泽布替尼（BGB-3111）：泽布替尼是我国自主开发的一种共价结合型高选择性BTKi。AU-003（NCT02343120）临床试验[13]–[14]是泽布替尼的首个Ⅰ/Ⅱ期临床试验。其中Ⅱ期部分入组22例TN CLL和101例R/R CLL患者以探索泽布替尼疗效和安全性。中位随访47.2个月，受试者的ORR分别为100％和94.6％，伴del（17p）和TP53突变的患者的ORR为87.5％，完全缓解（CR）率逐步增高。TN CLL的中位PFS未达，R/R CLL的中位PFS期为61.4个月。常见AE类型与伊布替尼接近，重点关注的房颤和高血压发生率持续保持较低，因AE导致用药中止的情况罕见。NCT03206918[15]是在我国开展的Ⅱ期单臂注册关键性临床试验，入组患者为R/R BineC期CLL或Ⅲ/Ⅳ期小淋巴细胞淋巴瘤（SLL），且有至少一项不良预后因素。中位随访15.1个月，ORR为84.6％，CR率为3.3％，各亚组之间无明显差异。安全性观察显示中性粒细胞减少发生率高，可能是由于泽布替尼更高的血药浓度和生物利用度造成。SEQUOIA[16]–[17]这项Ⅲ期临床试验将入组的TN CLL患者分为三组，A组和B组患者随机接受泽布替尼或B-R治疗以评估二者的差异，伴del（17p）的患者均进入C组接受泽布替尼治疗以评估泽布替尼治疗高危TN CLL的疗效。A组和B组对比结果明确表明，泽布替尼单药治疗疗效好于传统免疫化学治疗。C组全组ORR达到94.5％，CR率达到3.7％，表明泽布替尼治疗伴del（17p）的TN CLL疗效好，安全性高。

3. 奥布替尼（ICP-022）：奥布替尼也是我国开发的另一个共价结合型高选择性BTKi，2020年12月获批上市应用治疗R/R CLL[18]。Xu等[19]–[20]在我国组织开展的Ⅱ期开放标签多中心临床试验（NCT03493217）评估了奥布替尼治疗R/R CLL的疗效和安全性。研究中位随访时间32.3个月，ORR 92.5％，伴TP53突变或del（17p）患者的ORR为100％。CR率由中位随访时间14.3个月时的10.0％增至21.3％，证明治疗缓解深度持续加深。目前中位PFS和OS期均未达。安全性方面，多数AE为1～2级，没有患者出现房颤。入组患者中45例患者有既往HBV感染史即乙肝核心抗体呈阳性，其中33例患者在用药期间接受了抗乙肝病毒治疗，33例患者中仅1例出现乙肝病毒再激活。

上述临床试验结果表明，第二代共价结合型BTKi单药治疗CLL的BTK占有率显著提高，患者ORR较高，反应深度随治疗时间逐渐增加，且高危患者同样受益，较传统免疫化学治疗无论是疗效还是安全性均有显著优势。安全性方面，与第一代BTKi伊布替尼的AE种类基本相同，程度普遍较轻。重点关注的心血管事件发生率明显低于伊布替尼，因心血管事件中止治疗的情况罕见。对于因AE而无法耐受伊布替尼的患者，阿可替尼等可作为后续治疗选择。

二、第二代共价结合型高选择性BTKi与伊布替尼的非安慰剂对照临床试验

为明确第二代BTKi较伊布替尼在治疗CLL在药效以及安全性方面是否更具有优势，研究者开展了阿可替尼和泽布替尼与伊布替尼的非安慰剂对照Ⅲ期临床试验。

首先，Byrd等[21]组织开展了阿可替尼对比伊布替尼治疗R/R CLL的Ⅲ期ELEVATE-RR研究。入组533例R/R CLL且可伴del（17p）或del（11q）患者，1∶1随机分配接受阿可替尼或伊布替尼治疗（表2）。中位随访40.9个月，两组ORR接近，中位PFS期相同，阿可替尼的疗效数据达到了预设的非劣性指标。安全性方面，阿可替尼组因AE中止用药的患者例数少于伊布替尼组，心血管事件发生率和程度均低于伊布替尼，无患者因房颤中止治疗。

Ⅲ期开放标签的ALPINE[22]–[23]研究招募652例R/R CLL患者，1∶1入组接受泽布替尼或伊布替尼治疗。试验中位随访15.3个月和29.6个月时分别进行了中期和终期评估（表2）。两次评估的结果基本保持一致，泽布替尼在所有亚组中的ORR均高于伊布替尼，终期时伊布替尼的中位PFS期为34.2个月而泽布替尼中位PFS期未达，达到优效终点。安全性方面，泽布替尼组中因AE中止用药率始终低于伊布替尼组，泽布替尼中性粒细胞减少的发生率更高，但未导致感染发生率更高。重点关注的心脏毒性方面，两次评估中泽布替尼组因心血管事件中止治疗率和房颤发生率均低于伊布替尼组。

**表2:** 第二代BTK抑制剂与伊布替尼的非安慰剂对照比较研究[21]–[23]

试验名称	中位随访时间	分组	有效性					安全性	
			全部受试患者			伴del（17p）/TP53患者		常见≥3级AE发生率（%）	关注AE发生率（%）
			ORR	中位PFS期/PFS率（%）	中位OS期/OS率（%）	ORR	PFS率（%）		
NCT02477696	40.9个月	阿可替尼	77.0%	38.4个月	NR	NA	NA	中性粒细胞减少（19.5） 贫血（11.7） 肺炎（10.5）	房颤（9.4） 高血压（9.4） 出血（4.5）
	40.9个月	伊布替尼	81.0%	38.4个月	NR	NA	NA	中性粒细胞减少（22.8） 贫血（12.9） 肺炎（8.7）	房颤（16） 高血压（23.2） 出血（5.3）
ALPINE	15.3个月	泽布替尼	78.3%	1年（94.7）	1年（97.0）	80.5%	1年（91.5）	中性粒细胞减少（13.7） 高血压（10.3） 肺炎（3.9）	房颤（2.5） 出血（35.8） 高血压（16.7）
	15.3个月	伊布替尼	62.5%	1年（84.0）	1年（92.7）	50.0%	1年（74.4）	中性粒细胞减少（10.6） 高血压（7.2） 肺炎（4.8）	房颤（10.1） 出血（36.2） 高血压（16.4）
	29.6个月	泽布替尼	83.5%	2年（78.4）	NR	80.5%	2年（72.6）	中性粒细胞减少（16.0） 高血压（14.8） Covid-19相关肺炎（7.1）	房颤（5.2） 高血压（23.5）
	29.6个月	伊布替尼	74.2%	2年（65.9）	NR	55.3%	2年（54.6）	中性粒细胞减少（13.9） 高血压（11.1） 肺炎（4.9）	房颤（13.3） 高血压（22.8）

注 AE：不良事件；CR：完全缓解；NR：未达到；NA：暂缺；ORR：总缓解率；OS：总生存；PFS：无进展生存

上述的两项非安慰剂对照Ⅲ期临床试验结果证明第二代共价结合型高选择性BTKi的安全性尤其是降低心血管毒性明显优于伊布替尼。在疗效方面阿可替尼达到预设非劣性指标，表明其治疗CLL的效果与伊布替尼接近，而泽布替尼的疗效优于伊布替尼，ORR更高，有效延长受试者PFS期。以阿可替尼和泽布替尼为代表的第二代BTKi具有一定的治疗优势，已被NCCN等治疗指南推荐为CLL治疗的优先选择[24]。

三、第二代共价结合型高选择性BTKi联合治疗临床试验

尽管BTKi治疗CLL疗效显著，但单药治疗需终身用药，增加患者经济负担，药物累积毒副作用以及克隆选择导致耐药可能性。解决方案之一是BTKi联合其他药物以增加缓解深度，进行固定周期治疗。伊布替尼联合CD20单抗的临床试验取得了一定成绩，但结果未达预期，疗效上与单药治疗未见明显差异[25]。伊布替尼联合BCL2抑制剂方案在临床试验中展现了良好的安全性以及有限周期治疗的可行性[26]。以此为背景，目前多个第二代BTKi联合治疗的临床试验正在开展，部分临床试验已经取得一定成果。

阿可替尼联合奥妥珠单抗（acalabrutinib-obinutuzumab，A-O）的Ⅰb/Ⅱ期临床试验ACE-CL-003[27]纳入19例TN CLL患者和26例R/R CLL患者，TN CLL患者的ORR和CR率为95％和32％，R/R CLL患者分别是92％和8％，用药12个月后26％的TN CLL患者和15％的R/R CLL患者达到骨髓微小残留病阴性（undetectable minimal residual disease，uMRD）。该方案整体耐受良好，常见≥3级AE为中性粒细胞减少。阿可替尼联合奥妥珠单抗并没有使缓解深度明显增加，但无论是初治患者还是复发患者都有较高缓解率且耐受良好。

为进一步明确阿可替尼联合治疗、阿可替尼单药治疗以及传统免疫化学疗法在治疗上的差异，Sharman等[28]–[29]开展了Ⅲ期ELEVATE-TN试验，招募535例TN CLL患者，1∶1∶1随机分配接受阿可替尼联合奥妥珠单抗（A-O）、阿可替尼单药或奥妥珠单抗联合苯丁酸氮芥（obinutuzumab-chlorambucil，O-C）治疗。中位随访28.3个月和中位随访4年的临床数据表明含阿可替尼的治疗方案的缓解率和缓解深度均好于O-C组，A-O组缓解率最高，高危患者中差异更明显。O-C组中位PFS期为22.6个月，含阿可替尼的治疗组PFS均未达，A-O的预计两年PFS率高于单药治疗。含阿可替尼的治疗组治疗时间长，但因AE中止治疗率低于O-C组。该试验明确表明包含阿可替尼的治疗方案安全性和有效性均好于传统免疫化疗方案。受限于试验设计和人群，该试验无法证明联合治疗优于单药治疗，但结果表明相较单药治疗加入CD20单抗提高缓解深度并延长PFS期的同时增加感染、血细胞减少以及输液反应的风险。

两项Ⅱ期单臂治疗方案为阿可替尼联合维奈克拉（Venetoclax）和奥妥珠单抗的AVO固定周期试验和CLL2-BAG试验目前已取得初步结果。AVO固定周期试验入组63例TN CLL患者，中位随访27.6个月，ORR为100％，CR率逐渐增高，最高达46％。免疫球蛋白重链可变区（IGHV）基因和TP53的状态对CR率无明显影响。16周期后38％患者达到停药标准，25周期后剩余患者中51％达到停药标准，所有停药患者均无复发报道。CLL2-BAG试验入组45例R/R CLL患者，其中18例肿瘤负荷较高的患者在AVO治疗前增加了2周期苯达莫司汀减瘤治疗。6周期AVO治疗后ORR为100％，CR为18％，34例患者达到外周血uMRD，伴del（17p）的患者中64％达外周血uMRD。受试者中28％达停药标准。治疗后中位观察时间为13.8个月，2例患者出现Richter转化，无PD或死亡报道。两项试验均无新增安全事件，无药物累积毒性。CLL2-BAG中接受苯达莫司汀减瘤治疗的患者肿瘤裂解综合征以及输液反应的发生率显著降低。虽然两项试验达停药标准人数均未达到预期，但是结果表明AVO方案对TN CLL和R/R CLL患者均有治疗效果且耐受良好。AVO固定周期试验中16周期治疗后有大量患者达到较好PR且骨髓uMRD，CLL2-BAG中接受维持治疗的患者部分后续出现uMRD[30]–[31]。

临床前研究表明，泽布替尼可以增加线粒体对BCL2依赖性以及CLL细胞对BCL2抑制剂敏感性[32]。以此为背景开展的泽布替尼联合奥妥珠单抗和维奈克拉有限周期治疗Ⅱ期临床试验NCT03824483探索了泽布替尼联合治疗的疗效、安全性和停药可能性。入组39例高危TN CLL患者，中位随访25.8个月，ORR达100％，其中CR占57％，中位PFS期未达，89％的受试患者达到预设的外周血与骨髓uMRD并停药观察，1例患者终止后出现PD，恢复原方案治疗3个月后再次达到外周血uMRD。安全性上无新增AE，房颤发生率低，≥3级的中性粒细胞减少以及中性粒细胞缺乏伴发热的发生率要少于伊布替尼联合维奈克拉[33]。

上述临床试验结果表明第二代BTKi联合治疗CLL整体耐受性良好，适合包括有其他基础病史的高危患者以及曾接受过靶向药物治疗的患者，但会增加患者出现输液反应和肿瘤溶解的风险，可以考虑联合治疗前增加减瘤治疗。疗效上，ORR明显高于传统免疫化疗，较单药治疗反应深度增加，有部分受试者已达到停药目标，但比例未达预期。未达CR的患者MRD检测提示联合治疗停药后反应仍持续。这一现象也提出了针对固定周期治疗，相对于CR，以外周血或骨髓uMRD作为停药标准是否更符合疾病生理特征这一问题。第二代BTKi联合治疗的长期疗效以及安全性需进行长期的随访以及规模更大的临床试验。第二代BTKi联合治疗具有较高的研究价值，与单药或以伊布替尼为基础的联合治疗方案的非安慰剂对照临床试验值得探索。除此之外，奥布替尼联合氟达拉滨、环磷酰胺和奥妥珠单抗的cwCLL-001研究（NCT05322733）已在国内开展并招募患者。BTKi联合PI3Kδ抑制剂Zandelisib以及PDL-1单抗度伐利尤单抗（Durvalumab）的相关临床试验也正在积极开展，有望拓宽固定周期治疗的药物选择范围。

四、可逆非共价结合BTKi与BTK靶向蛋白降解嵌合体的研究进展

针对共价结合BTKi单药治疗出现的耐药问题，目前可逆非共价结合BTKi以及BTK PROTAC两种药物提供了解决思路。可逆非共价结合BTKi通过与BTK的ATP结合位点非共价结合避开传统的BTK C481位点来达到治疗目的的同时克服结合位点突变造成的耐药问题，目前已有Pirtobrutinib（LOXO-305）和MK-1026（ARQ-531）进入临床试验阶段。Pirtobrutinib的Ⅰ/Ⅱ期BRUIN试验[34]参与药效评估的受试者包括139例CLL/SLL患者，其中121例有共价结合BTKi治疗经历。结果表明LOXO-305无剂量相关毒性，最常见的≥3级AE是中性粒细胞减少。接受过共价结合BTKi治疗患者ORR为62％，其中因BTK结合位点突变中止的患者ORR达75％，且发现随着治疗的推进，对药物有反应的患者的外周血单核细胞BTK C481突变丰度逐渐降低。研究结果表明LOXO-305治疗CLL安全有效，并且适用于多次治疗或对共价结合型BTKi已经产生耐药的CLL的患者。由Woyach等[35]组织开展的MK-1026（ARQ-531）Ⅰ期临床试验已有初步结果，证实该药在治疗接受过多次治疗的CLL患者时耐受性良好，有明确抗肿瘤活性。目前Ⅱ期临床试验（NCT04728893）正在积极招募受试患者。

靶向蛋白降解嵌合体（PROTAC）是新出现的一种可以选择性降解细胞内蛋白质的化学方法。作用机制是PROTAC分子将目标蛋白与E3连接酶远端连接，利用真核细胞的泛素-蛋白酶体途径，诱导继发目标蛋白降解。以此为背景，将BTK作为目标蛋白研发的BTK PROTAC有望降解BTK，从根源上治疗CLL。目前已有如P13I [36]、NX-2127、BGB16673和HSK29116等药物成功合成并在临床前研究中成功降解CLL细胞的BTK。NX-2127的首个临床试验NX-2127-001（NCT04830137）在第64届美国血液学会年会（ASH）上公开了部分数据。入组17例R/R CLL患者均接受过BTKi治疗，其中76.5％还接受过维奈克拉治疗，部分为 BTKi和BCL2抑制剂双耐药患者，以及可逆非共价结合BTKi治疗后进展患者。用药第一周期的中位BTK降解率为83％，ORR为33％，且观察到ORR随用药时间延长逐步增加。值得注意的是，在BTKi和BCL2双耐药以及可逆非共价结合BTKi治疗后进展患者中也观察到了药物反应。这些结果为后续的临床研究提供了支持[37]。我国研发的BTK PROTAC HSK29116的临床试验NCT04861779正在积极招募患者进行安全性、耐受性以及药物动力学研究。

目前相关临床试验目前随访时间较短，仍需长期随访数据评估药物安全性与反应的持久性。两种治疗机制为共价结合BTKi治疗后出现的耐药问题带来了治疗思路，甚至有望达到根治CLL。后续可探索与共价结合BTKi或传统免疫化疗的非安慰剂对照临床试验以及联合治疗可能性。

五、总结

BTKi越来越广泛的临床应用，使CLL患者治疗可选择的方案越来越多。尽管目前CLL仍不可治愈，但患者预后和生存状况显著改善。以阿可替尼、泽布替尼和奥布替尼为代表的第二代BTKi可以弥补伊布替尼疗效和安全性上的不足，但对造血系统的严重影响以及单药长期治疗无法停药的问题，仍需关注长期随访结果。BTKi联合CD20单抗以及BCL2抑制剂为代表的有限周期治疗研究结果令人鼓舞，同时提出了评估CLL治疗效果以及停药标准的方法是否需要重新衡量的问题。针对耐药问题研发的可逆非共价结合BTKi以及BTK PROTAC均值得后续继续关注研究进展。
